# Rapid Development of Adaptive, Climate-Driven Clinal Variation in Seed Mass in the Invasive Annual Forb *Echium plantagineum* L.

**DOI:** 10.1371/journal.pone.0049000

**Published:** 2012-12-19

**Authors:** Tara K. Konarzewski, Brad R. Murray, Robert C. Godfree

**Affiliations:** 1 Plant Functional Biology and Climate Change Cluster, School of the Environment, University of Technology Sydney, New South Wales, Australia; 2 CSIRO Plant Industry, Canberra, Australia; Norwegian University of Science and Technology, Norway

## Abstract

We examined adaptive clinal variation in seed mass among populations of an invasive annual species, *Echium plantagineum*, in response to climatic selection. We collected seeds from 34 field populations from a 1,000 km long temperature and rainfall gradient across the species' introduced range in south-eastern Australia. Seeds were germinated, grown to reproductive age under common glasshouse conditions, and progeny seeds were harvested and weighed. Analyses showed that seed mass was significantly related to climatic factors, with populations sourced from hotter, more arid sites producing heavier seeds than populations from cooler and wetter sites. Seed mass was not related to edaphic factors. We also found that seed mass was significantly related to both longitude and latitude with each degree of longitude west and latitude north increasing seed mass by around 2.5% and 4% on average. There was little evidence that within-population or between-population variation in seed mass varied in a systematic manner across the study region. Our findings provide compelling evidence for development of a strong cline in seed mass across the geographic range of a widespread and highly successful invasive annual forb. Since large seed mass is known to provide reproductive assurance for plants in arid environments, our results support the hypothesis that the fitness and range potential of invasive species can increase as a result of genetic divergence of populations along broad climatic gradients. In *E. plantagineum* population-level differentiation has occurred in 150 years or less, indicating that the adaptation process can be rapid.

## Introduction

Successful invasion of novel environments by exotic plant species requires that species maintain positive population growth and spread in the face of environmental heterogeneity and new selection pressures [1], [2]. While many factors determine the demographic characteristics and spatial spread of invading plant populations [3], rapid evolutionary changes in fitness-related traits increase the reproductive output of local populations and often play a fundamental role in the invasion process [1], [4], [5]. Indeed, significant evolutionary capacity has been identified in many invasive plant species [6]–[12]. This is perhaps not surprising, since considerable theoretical and empirical evidence supports the notion that the capacity for rapid evolutionary change exists widely in plant populations (e.g., [1], [13]–[15]).

Recently it has been suggested that, at large spatial scales, the spread of invasive populations is mainly determined by evolutionary adaptation and population-level genetic differentiation, while phenotypic plasticity becomes more important where small-scale variation in abiotic conditions impact on population fitness [16]. While the adaptive importance of phenotypic plasticity is well understood [17]–[21], the capacity for invasive species to undergo adaptive differentiation along broad-scale climatic gradients has been more poorly documented. Adaptive clinal variation in life-history traits has been observed in some invasive species (e.g., [6],[9]–[12],[20],[22]), and their ability to occupy new climatically distinct envelopes in their introduced range is likely to be a valuable strategy in general [23].

However, not all invasive species display clinal differentiation [24],[25], perhaps due to the wide range of genetic, demographic, developmental and environmental factors that influence evolutionary divergence of plant populations in new habitats [1],[26]–[29]. Peripheral populations located in marginal habitats, for example, suffer numerous evolutionary constraints related to population size, gene flow and migration rates [30]–[32]. Levels of phenotypic plasticity [19], seed dormancy [33], co-variation among fitness traits [29], and pathogen load [34] are also known (among other factors) to limit evolutionary adaptation, and many are especially relevant for invasive plant populations. Given this conflicting evidence, there is a clear need for a more comprehensive understanding of the species, circumstances, and traits in which adaptive clines are likely to develop.

Seed mass is a key fitness-related trait that might be expected to show strong clinal adaptation when the ability of a species to produce seeds of a particular size underpins reproductive success and survival in new environments [35],[36]. Seed mass influences many life-history traits including dispersal ability, seed bank viability and persistence, progeny fitness, flower size and plant longevity [37],[38]. Large seed size appears to be especially important in arid zone species probably due to the increased temperature-related metabolic costs and requirements for seedling establishment in arid environments [35],[39],[40]. Evolution of seed mass in response to environmental gradients is indeed well documented on a local [40]–[45] and global scale [46],[47] although few studies have considered whether such patterns exist among invasive species (but see; [46],[48],[49]).

The aim of this study was to test whether, over the past ∼150 years since introduction, invasive populations of the annual plant species *Echium plantagineum* L. (Paterson's curse) have developed adaptive, population-level differentiation in seed mass in response to broad climatic gradients in south-eastern (SE) Australia. *Echium plantagineum* is a genetically diverse [50], globally significant weed [51]. In Australia, it has invaded arid, temperate and coastal environments, costing the meat and wool industry upwards of $125 million annually [52]. We hypothesized that invasive populations of *E. plantagineum* have developed a cline in seed mass in response to aridity, with larger seeds prevailing in populations sourced from warmer, drier habitats than in those sourced from cooler, wetter temperate and coastal habitats. We also hypothesized that ongoing selection for seed size will have resulted in a narrowing of seed size variation among populations within bioregions and among individual plants within populations in the most arid and unfavourable environments relative to populations from a more favourable core habitat [29],[30]. To test these hypotheses, we compared the weights of glasshouse-produced seeds from invasive *E. plantagineum* populations sourced from 34 sites across a very large (1,000 km) temperature and rainfall gradient in SE Australia.

## Methods

### Study species

**Figure 1:**
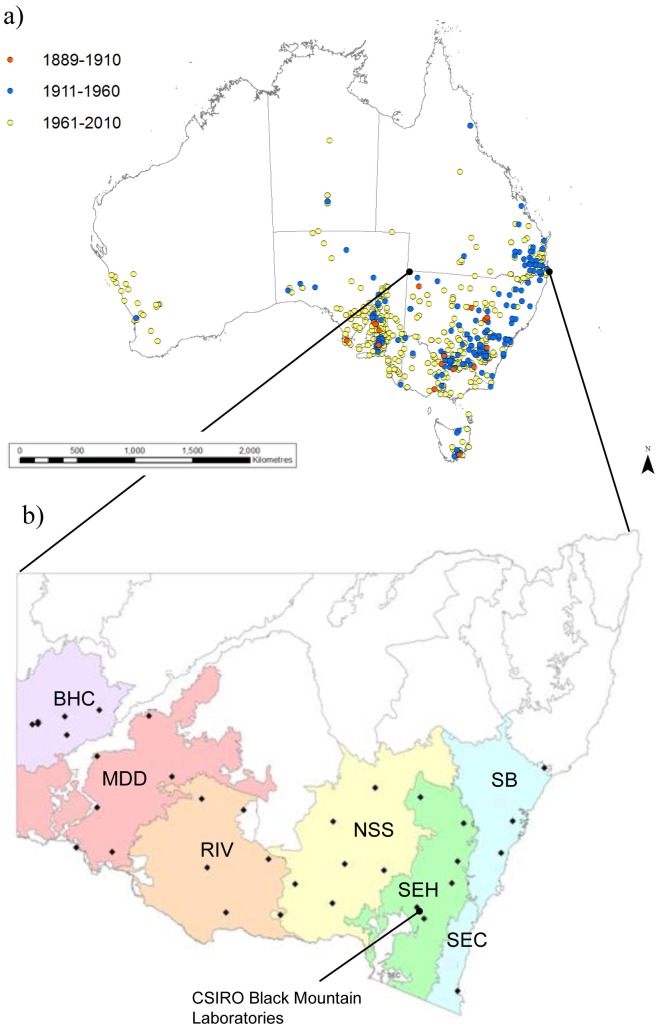
Distribution of *Echium plantagineum* in Australia and location of collection sites across south-eastern Australia. (a) Herbarium records from the periods 1889–1910, 1911–1960 and 1961–2010 based on data obtained from Australia's Virtual Herbarium (2009 Council of Heads of Australasian Herbaria Inc) show the pattern of spread since introduction. (b) Distribution of study sites (depicted by black diamonds) across south-eastern Australia, grouped by IBRA bioregion (coloured). BHC = Broken Hill Complex, MDD = Murray Darling Depression, RIV = Riverina, NSS = NSW (New South Wales) South-Western Slopes, SEH = South-Eastern Highlands, SB = Sydney Basin, SEC = South-East Corner. Note that for all analyses SB and SEC were combined into a single coastal bioregion (COAST). The location of CSIRO Black Mountain Laboratories (S 35.27°, E 149.12°) where the glasshouse experiment was conducted is indicated.

Originally native to Europe and the Mediterranean region, *Echium plantagineum* (Boraginaceae) is an annual forb that was introduced to Australia in around 1850 [51]. It is a globally invasive species that has become successfully established in 30 million hectares of agricultural land in Australia [53],[54] (Fig. 1a). *Echium plantagineum* is insect pollinated and can produce up to 10,000 seeds with seed production of up to 30,000 per m^2^. Seeds are dispersed via water, contaminated fodder, garden waste, animal fur and the alimentary tracts of birds or grazing animals [54],[55], and while some seeds can remain dormant in the soil for up to ten years [55],[56], most germinate more rapidly [51]. Seedlings most effectively colonise bare ground [57] and can have recruitment rates of >1,000 m^−2^ [56]. In annual species like *E. plantagineum* frequent seed production is a critical driver of population fitness, making it an ideal model species for studies of adaptive capacity and evolutionary responses of seed-related traits in response to broad-scale climatic variation.

### Seed collection and field sites

*Echium plantagineum* seeds were collected during the 2009 reproductive season (October to December) from a total of 34 sites across seven IBRA (Interim Biogeographic Regionalisation for Australia scientific framework; [58]), bioregions in New South Wales, SE Australia (Fig. 1b). These bioregions are large, geographically distinct areas of land with similar climate, land systems, vegetation and animal communities [58]. The study sites followed a 1000 km long climatic cline which varies from arid (Broken Hill Complex bioregion) to coastal (Sydney Basin and South-East Coast bioregions) and cool temperate (South-East Highlands bioregion; Fig. 1b, Table 1). These bioregions capture a large majority of *E. plantagineum* habitats in SE Australia. Due to similarities between the two coastal bioregions (Sydney Basin and South-East Coast; Fig. 1b) and the limited number of sites containing *E. plantagineum*, these bioregions have been combined to form a single coastal bioregion (COAST) for our analysis. Sites were randomly selected from across each bioregion; all had at least 50 seed-producing plants. Seeds were collected from ten randomly selected individual plants at each site between October 2009 (Broken Hill Complex bioregion) and December 2009 (COAST bioregion), when plants were producing mature seeds. No permits were required for the field collections since *E. plantagineum* is an introduced, invasive species. No collections were made on private land. Seeds were transported to the laboratory (CSIRO Black Mountain Laboratories, Canberra, ACT; Fig. 1b), extracted from the mature fruit using a rubbing board (consisting of two flat rubber pads), and stored in paper bags at room temperature until used in the following glasshouse experiment.

**Table 1:** Environmental characteristics of the six bioregions from the Interim Biogeographic Regionalisation for Australia Framework (IBRA) sampled within the study region.

Bioregion^1^	Elevation (m)	Precipitation (mm)^2^	Tmax (°C)^2^	Tmin (°C)^2^	Tav (°C)^2^	PET (mm)^2^	AWB (mm)^2^	Aridity Index^2^	pH (CaCl_2_)^3^	Electrical conductivity (µs/cm)^3^	Nitrogen %^3^	Carbon %^3^
BHC	266	109	19.7	7.5	13.6	513	−805.9	0.21	8.1	142	0.10	2.3
MDD	109	141	19.9	6.9	13.4	470	−656.1	0.30	7.9	130	0.16	3
RIV	156	202	18.4	6.1	12.2	429	−452.4	0.48	6.8	187	0.22	2.6
NSS	353	273	17.1	5.1	11.1	386	−224.3	0.71	6.0	202	0.16	1.9
SEH	882	383	13.5	2.7	8.1	338	91.6	1.14	6.2	73	0.17	2.2
COAST	178	358	18.9	7.1	13.0	419	−121.8	0.86	5.6	177	0.48	6.3

1 BHC = Broken Hill Complex; MDD = Murray Darling Depression; RIV = Riverina, NSS = NSW South-Western Slopes, SEH = South-Eastern Highlands, COAST = combined Sydney Basin and South-East Corner bioregions. Bioregion means are averages of site-level data within each bioregion.

2 Mean climatic data (1910–2010) were determined for the May to October growing season (see methods).

3 Edaphic factors were measured during October–December 2009.

Tmax = maximum temperature, Tmin = minimum temperature, Tav = average temperature, PET = potential evapotranspiration, atmospheric water balance (AWB) = precipitation – PET [64], Aridity Index = precipitation/PET [65].

### Common garden glasshouse experiment

The objective of the glasshouse experiment was to compare the mass of seeds produced by different plants under common growing conditions, thus allowing for a more controlled assessment of the genetic basis of existing variation. Experimental maternal effects were minimised by using seeds from different populations that were equivalent in mass, size, germination time and level of dormancy. Seed choice was facilitated by the fact that field-collected seeds from different bioregions did not differ in mass (TK Konarzewski unpublished data), unlike glasshouse-produced seeds (see below). This probably reflects the dry conditions experienced during the collection year (2009), especially in the most westerly bioregions, since drought stress in reproductive plants can reduce seed mass [59] and cause general divergence of plant traits under field and glasshouse conditions [22],[42]. Nonetheless, all populations produced large numbers of viable, fully mature seeds which were adequate for experimental use.

Ten seeds from each of ten plants from each site (3400 seeds in total) were germinated in the laboratory on moist filter paper in petri dishes under dark conditions at room temperature. After the radicle had emerged, one similar sized embryo (based on radicle length) from each plant (340 in total) was transplanted into small biodegradable pots (Jiffypot®) in a temperature controlled glasshouse at the CSIRO Black Mountain site. After ten days the pots were planted into 10 cm pots of standard potting mix, and then four weeks prior to the commencement of the experiment, plants were again transplanted into 20 cm pots containing standard, high nutrient compost potting soil (consisting of a mix of calcium carbonate lime, dolomite lime, blood and bone, and NPK fertiliser; pH = 6.5). Pots were arranged in a randomised block design with five blocks each consisting of three benches; two plants from each of the 34 study sites were randomly placed in each block. Plants were grown from April to December 2010 under a photoperiod governed by natural sunlight and a targeted day/night temperature regime of 25/15°C with an average of 20°C. Temperatures were logged from August to October and followed the targeted regime reasonably closely, with daily averages of 16–20°C, although spot temperatures as high as 27°C and as low as 12°C were observed. Of the 340 plants used in the experiment, two died and 34 plants did not flower within the duration of the experiment (250 days); these were removed from further consideration. After 27 weeks sufficient flowers were produced to allow pollination. Pots were fertilised with Aquasol® Soluble Fertiliser (Yates, Australia) fortnightly or as required.

Suitable plants for open pollination were defined as plants with ten or more open receptive healthy flowers which were identified by the shape and size of the flower and the length and maturity of the stigma. Between October and November 2010 plants from each site were transported together but separately from plants from other sites to a pollination chamber (a small naturally-lit glasshouse) to ensure that cross pollination occurred among plants that originated from the same site. Between six and ten plants, with suitable numbers of flowers, were available for each site. Plants were stored in a separate, insect-free glasshouse for 24 hours prior to placement in the pollination chamber and previously mature flowers were removed to ensure that only newly developed flowers were pollinated. Pollination was performed by European honey bees (*Apis mellifera*) with an exposure period of 24 hours. Plants were changed at night when the bees returned to their hive to reduce the risk of cross pollination between sites. All plants were then moved back to the glasshouse (which was also insect free) to complete their development. Seeds were collected from all plants after five weeks following seed maturation and placed into paper bags for storage at room temperature. Seed production was only observed in flowers that were exposed to bee pollination.

### Seed measurements

Seed mass per individual plant was determined as the weight of ten viable seeds dried for one week at a temperature of 80°C, expressed as seed weight (g) per 100 seeds. Seed viability was determined by visual inspection and lightly pressing on either side of the seed with forceps. Experience with germination of field collected seeds indicated that seeds were viable if the seed coat did not crack or deform under light pressure (i.e., the seed was filled)

### Site characterisation

One representative soil core, 10 cm^2^ and 10 cm deep, was collected at each field site, sealed in a plastic bag, and stored at room temperature in the laboratory. A representative subsample (five grams) was ground to a fine powder using a tissuelyser at a frequency of 30.1 rpm for ten minutes, and analysed for percentage carbon (C) and percentage nitrogen (N) using a Europa 20–20 isotope ratio mass spectrometer with an automated nitrogen carbon analysis preparation system [60]. Soil electrical conductivity and soil pH (CaCl_2_) were measured as described in [61].

Climate data for the period 1910–2010 were obtained for all study sites using the SILO enhanced meteorological dataset and datadrill procedure hosted by The State of Queensland (Department of Environment and Resource Management) 2012 (see http://www.longpaddock.qld.gov.au/silo/ [62]). We then derived five key climatological variables for each site, focusing on the time frame of May to October during which *E. plantagineum* growth and effective precipitation are highest [63]: total precipitation (P), mean maximum temperature, mean minimum temperature, mean temperature, and total potential evapotranspiration (PET). We also derived two measures of aridity for each site: 1) the annual atmospheric water balance (AWB) [64], defined as AWB = P- PET and 2) the aridity index (AI) [65], defined as AI = P/PET (See Table 1 for summary).

**Figure 2:**
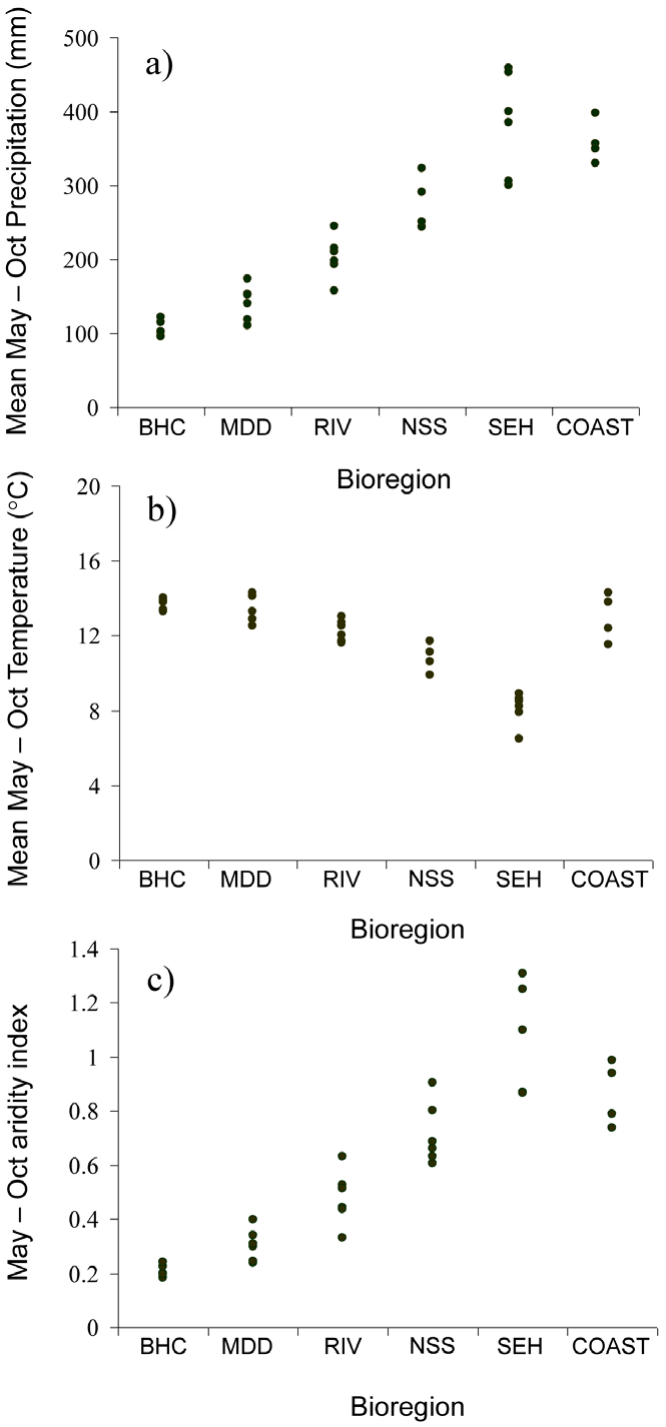
Climatological data for the May–October growing season for bioregions in the study area. Data are based on 1910–2010 averages derived from the enhanced meteorological dataset hosted by The State of Queensland (Department of Environment and Resource Management) 2012 (http://www.longpaddock.qld.gov.au/silo/). (a) Total precipitation (mm). (b) Mean temperature (°C). (c) Aridity Index. Abbreviations denote the individual bioregions, See Fig. 1 for full names. Bioregions are arranged along the *x* axis from most westerly (BHC) to most easterly (COAST).

Mean climatological conditions during the historical (1910–2010) May–October *Echium* growing season for each bioregion are shown in Table 1. Total precipitation increases from around 100 mm in the arid Broken Hill Complex to >300 mm in COAST and South-East Highland bioregions (Fig. 2a). Higher bioregions in the east (e.g., NSW South-West Slopes and South-East Highlands) experience cooler temperatures than coastal or far inland locations (Fig. 2b). The combination of increasingly drier and warmer conditions towards the west of the study region results in a sharp increase in aridity from the South-East Highland to Broken Hill Complex bioregions (Fig. 2c); aridity is intermediate in coastal habitats due to the high overall rainfall.

### Statistical analyses

The primary data set consisted of mean seed mass for 189 seed-producing plants across 34 study sites (the remainder either died, produced no flowers, or produced no viable seeds). We constructed the final data set for analysis by removing data for the parental plants (n = 45) that produced fewer than three seeds since in most cases the seeds produced were very small due to early abortion or senescence of the fertilised flowers. For one site we retained data from a single plant that produced two healthy, viable seeds because it was the only datum available for that site. The final data set thus consisted of mean seed mass for 144 seed-producing plants. While we report the results of analyses conducted on the final data set, because the presence of small or aborted seeds may reflect varying levels of self incompatibility or inbreeding depression [66], we conducted all analyses on both data sets. This decision had no impact on interpretation of the results, although the exclusion of smaller seeds did slightly reduce among-site variation in seed mass.

Linear mixed model analysis was used to relate seed mass to bioregion (fixed predictor variable), site within bioregion (random) and block (fixed). Effects of fixed variables were tested using standard F-tests and the effect of the random variable was tested using the Wald Z test [67]. The final seed mass data set was square-root transformed according to *y* = sqrt(*x*) to meet model assumptions. Post-hoc tests were performed on bioregion means using the Tukey-Kramer adjustment for multiple testing [68].

**Figure 3:**
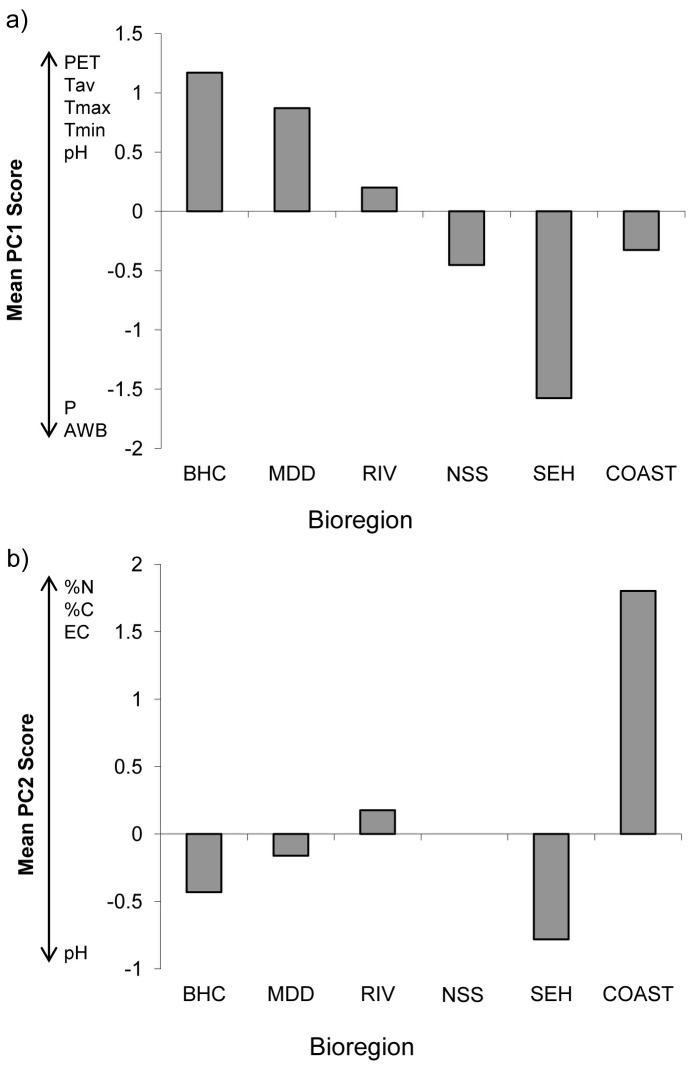
Mean scores for each bioregion on first and second principal components derived from PCA of data from ten climatological and soil-related variables collected at each of the 34 study sites (see **methods** **).** a) Mean scores on PC1. b) Mean scores on PC2. Variables with high (>0.40) loadings on PC1 and PC2 are shown beside the *y*-axis; upward and downward arrows indicate positive and negative loadings respectively. For example, in (a) average annual temperature (Tav) loads positively on PC1 while atmospheric water balance (AWB) loads negatively. The data show that PC1 is primarily related to overall aridity, which increases from the most mesic (South-Eastern Highlands) to most arid (Broken Hill Complex) bioregions, while PC2 primarily distinguishes between COAST and South-Eastern Highland bioregions based on soil characteristics. Abbreviations denote the individual bioregions; see Fig. 1 for full names. Bioregions are arranged along the *x* axis from most westerly (BHC) to most easterly (COAST). Climate and soil variable acronyms defined in the text except %N = percentage soil nitrogen; %C = percentage soil carbon; EC = soil electroconductivity.

We also quantified the direct relationships between seed mass and specific environmental characteristics of maternal field site using linear regression. We first used principal component analysis (PCA) to reduce the ten correlated environmental variables describing the sites (six climatic, four soil variables; see Table 2) to two components that, combined, accounted for 82% of the total variance. The first component (PC1) accounted for 58% of the variation in the data and was strongly associated with climatic variables (Table 2). Scores on PC1 decrease from the arid (BHC) to mesic (SEH) bioregions, with coastal, slopes and Riverina regions having intermediate scores (Fig. 3a). The second component (PC2) accounted for 24% of the variation and primarily reflected site-level soil characteristics (Table 2). PC2 primarily distinguished between coastal and highland bioregions (Fig. 3b), with low-elevation coastal areas having higher soil fertility (Table 2). The relationship between soil pH and PC1 (Table 2; Fig. 3a) is indicative of the general tendency for soil acidity to increase from the western to eastern parts of the study area [69], although soil pH also loaded on PC2.

**Table 2:** Component loadings of ten variables based on principal components analysis of site-level climatic and soil data.

Variable^1^	PC 1^2^	PC 2^2^
Atmospheric water balance	**−.972**	.047
Potential evapotranspiration	**.957**	.065
Precipitation	**−.931**	.175
Maximum temperature	**.924**	.290
Average temperature	**.923**	.317
Minimum temperature	**.895**	.345
Soil pH	**.763**	**−.406**
Soil nitrogen	−.070	**.957**
Soil carbon	.062	**.891**
Soil electrical conductivity	.100	**.474**

1 Climatic data for the May to October growing season (1910–2010) (see methods). Potential evapotranspiration (PET); atmospheric water balance (AWB) = precipitation- PET.

2 Component loadings above 0.400 or below – 0.400 are in bold.

Both PC1 and PC2 were related to mean site-level seed mass using linear regression analysis. Finally, we used individual linear regression analyses [67],[70] to directly assess the impact of latitude and longitude on mean site-level seed mass since these relationships have been previously assessed for a number of species in Australia (e.g., [71]). Mean growing season (May–October) precipitation, temperature, and aridity index were also used in individual regression analyses in order to directly determine the relationships between these variables and seed mass. No data transformations were required for the regression analyses.

We next tested whether site-level seed mass variance differed across bioregions. First, we performed Levene's test of homogeneity on non-transformed data to determine whether variance among populations differed among bioregions. We then performed a one-way analysis of variance to determine whether mean among population seed mass variance differed across bioregions. Data from two sites were excluded because plants produced insufficient seeds to determine variance. Finally, we used simple linear regression to determine whether among population seed mass variance was related to longitude, latitude and scores on PC1 and PC2. All statistical analyses were performed using SAS version 9.1 (SAS Institute Inc., Carey, NC, USA).

## Results

**Figure 4:**
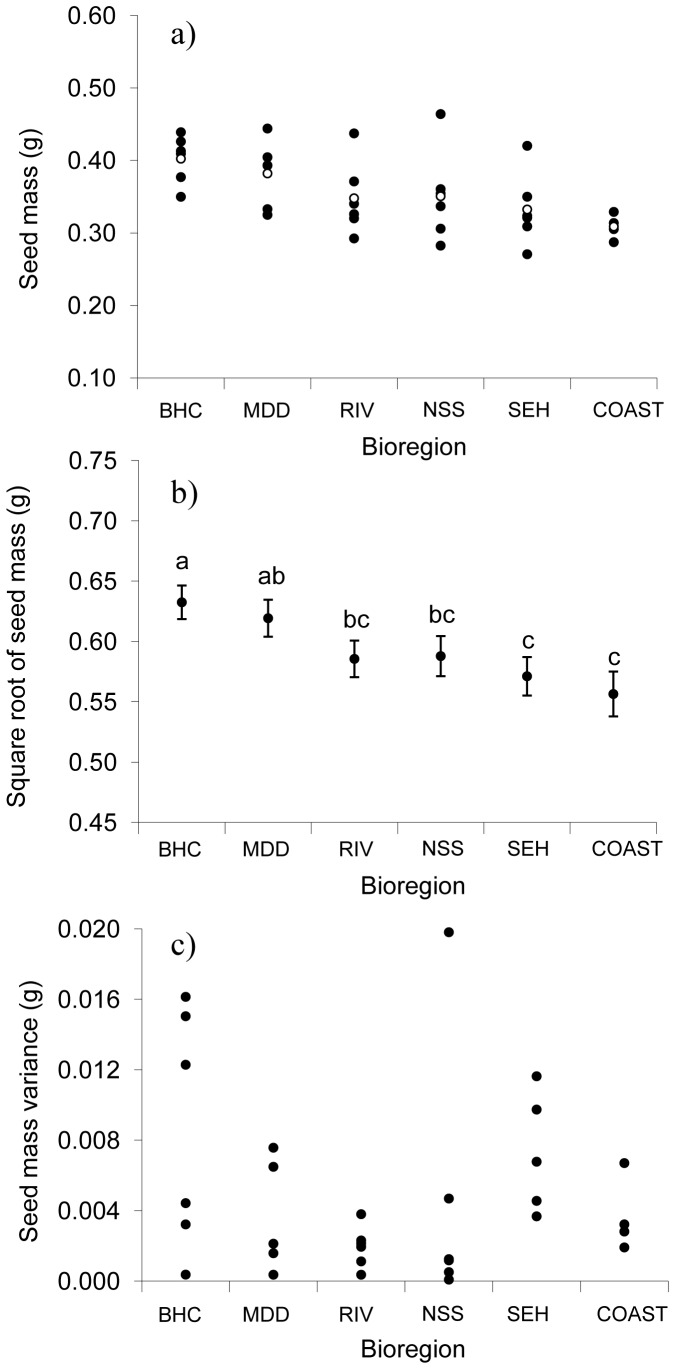
Seed mass and seed mass variance derived from plants collected from six bioregions in the study area. (a) Mean site-level seed mass (g; 100 seeds) across all six bioregions based on the final data set (with small seeds removed). Site means are shown as filled circles while bioregion means (average of all sites within a bioregion) are shown as unfilled circles. (b) Estimated mean seed mass (±1 SE) for each bioregion based on linear mixed model analysis (see methods) of final data set (square root transformed). Means sharing the same letter did not differ significantly at the 0.05 level. (c) Variance in seed mass for all study sites in each bioregion, determined as the variance in seed mass among seed-producing plants using the final data set. Abbreviations denote the individual bioregions, See Fig. 1 for full names. Bioregions are arranged along the *x* axis from most westerly (BHC) to most easterly (COAST).

Seed mass varied significantly among bioregions (F_(5,28)_ = 3.42, P = 0.02, Fig. 4a,b), but not among sites within bioregion (WALD Z = 1.20, P = 0.12). Seed mass did not vary significantly across blocks (F_(4,106)_ = 0.77, P = 0.54). There was an overall pattern for seeds sourced from populations found in drier bioregions (especially the Broken Hill Complex and Murray Darling Depression) to be heavier than those sourced from more mesic coastal and SE highland habitats (Fig. 4a,b). Mean seed weight in Broken Hill Complex populations was 23% higher than that of COAST populations (Fig. 4a), with seeds produced by plants from semi-arid (Riverina) and temperate (NSW South-Western Slopes, South-Eastern Highlands) bioregions being intermediate in weight (Fig. 4a).

**Figure 5:**
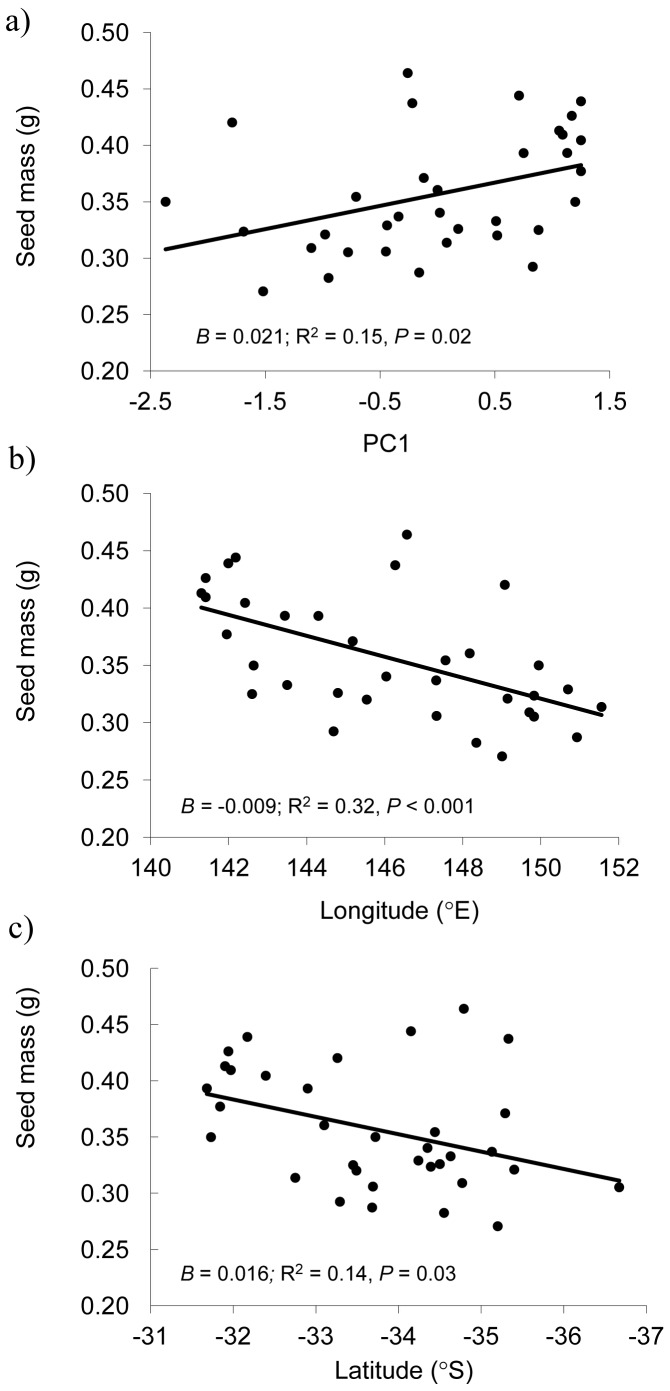
Scatterplots depicting linear relationships between seed mass and three key predictor variables: a) site-level scores on PC1 (primarily related to climate, see **Table 2**), b) site longitude, and c) site latitude. *B* = unstandardised regression coefficient.

At the population level, seed mass was significantly related to PC1 (Fig. 5a) but not PC2 (*B* (unstandardised regression coefficient) = −0.012, R^2^ = 0.05, P = 0.19), indicating that climatic factors were more important than soil-related factors, in determining variation in seed mass. Seed mass was strongly related to longitude (Fig. 5b). Each degree of longitude reduced predicted 100-seed weight by around 0.01 g (from a maximum of ∼0.40 g in the Broken Hill Complex to a minimum of ∼0.30 g in the COAST bioregion; Fig. 5b). This represents a decline of, on average, ∼2.5% per degree of longitude. Seed mass also declined with May–October rainfall (1910–2010) (*B* = −0.0002, R^2^ = 0.18, P = 0.01), aridity index (*B* = −0.058, R^2^ = 0.15 , P = 0.02) but only marginally with mean temperature (*B* = 0.007, R^2^ = 0.09, P = 0.09). Finally, seed mass was also significantly related to latitude (Fig. 5c), with each degree of latitude reducing predicted mean seed mass by 0.016 g, or around 4% (on average over the entire study region).

Variance among populations within bioregion did not differ significantly (Levene's test of homogeneity on untransformed data F_(5,26)_ = 1.36, P = 0.27; Fig. 4a), and mean site-level variation in seed mass (calculated as the variance in seed mass among plants within each site) did not differ significantly across bioregions (F_5,26_ = 1.63, P = 0.19; Fig. 4c). There was a general trend for between-site variance in seed mass (Fig. 4a) to be greatest towards the core of the species distribution (e.g., NSW South-Western Slopes and South-Eastern Highland bioregions) and least towards the edge (e.g., Broken Hill Complex and COAST bioregions). This result was not observed in among-plant variation at the site level (Fig. 4c). Site-level variation in seed mass was also not related to longitude, latitude, or scores on PC1 or PC2 (P>>0.05 for all).

## Discussion

The results of this study support the hypothesis that relatively recent climatic adaptation has resulted in the development of a cline in *Echium plantagineum* seed mass with aridity in SE Australia. Our data indicate that *E. plantagineum* seed mass declines by around 25% across the 1,000 km west-east gradient spanning the study area, with smaller seeds being produced by populations from cooler, wetter environments (e.g., COAST bioregion) than warmer, drier environments (e.g. Broken Hill Complex bioregion). Seed mass correspondingly declines by close to 2.5% (on average) per degree of longitude, and broadly increases with site aridity, which is highest in the west of the study region. We also found that seed mass declines with increasing latitude, although the strength of the association is lower than for longitude (Fig. 5b,c).

These data suggest that selection pressure, associated with aridity, has acted on invasive populations of *E. plantagineum* to increase seed size in arid relative to mesic habitats. This supports the general argument that seed mass plays an important role in maintaining population fitness in arid-adapted species [35],[39], and that seed-related traits have the capacity to undergo evolutionary shifts that increase the invasive potential of newly introduced plant populations [45]. Adaptive variation in seed mass in response to broad environmental gradients has been well documented [40]–[45],[47]; but this study is one of the first to document such a change in an invasive species (see [46],[48],[49] for other examples).

Interestingly, the decline in seed mass of 2.5% per degree of longitude and 4% per degree of latitude in *E. plantagineum* is remarkably similar to that reported by [71] for native perennial *Glycine* species in Australia. They attributed the presence of larger seeds in inland areas and at low latitudes to the increased metabolic costs of high temperature and increase in availability of photosynthate in these environments [40], which is a plausible explanation for existence of the same pattern in *E. plantagineum*. The high degree of spatial convergence of *Echium* and *Glycine* strongly supports the view that, in Australia, both native and exotic species experience similar climatic selection pressures and have the capacity to develop adaptive clinal variation in seed mass in response. However, while seed mass evolution is likely to have increased the overall distribution, abundance and fitness of *E. plantagineum*, other factors, such as overall seed size [45], broad differences in other life history traits, or the effects of disease [34] are also likely to play a key role in determining the performance of this and other invasive species relative to sympatric native species.

We also hypothesized that variation in seed mass should decrease in populations subjected to the strongest directional selection pressure (see [29]), which in this case is more arid areas where seed mass is known to be a key determinant of fitness [35],[39]. However, in contrast to mean seed mass, we did not find a clear pattern in seed mass variation across the study region. Variation in seed mass among plants at the individual site scale was unrelated to longitude, latitude, or variation in climate and soil (i.e., PC1), and did not differ across bioregions (Fig. 4c). This is consistent with other studies that have shown *E. plantagineum* populations in Australia to be extremely genetically diverse, with geographically isolated peripheral populations as diverse as those located in core habitat [50],[72]. It is worth noting, however, that the magnitude of variation across different plants within each study site was relatively low (coefficients of variation mainly in the 10–30% range; data not shown), and could reflect experimental error. Another possibility is that other processes, including gene flow and resulting migration-selection balance [30],[31],[73], genetic drift in peripheral populations with small effective population sizes [74], increased local genetic diversity arising from genotypic admixture [30] or “genetic rescue” [31] could have resulted in the lack of clinal development in variation for seed mass.

Variation among sites in seed mass also did not differ among bioregions, but there was a suggestion that sites had greater similarity in mean seed size (i.e., lower variance) at either ends of the cline than in the centre (Fig. 4a). This effect was more evident in site means determined from the full data set. The overall relationship between seed mass variance and clinal location (e.g. longitude) appears to be curvilinear, with a peak in the NSS bioregion, and if true, could reflect the effects of directional selection operating more strongly on heritable genetic variation in marginal than in core populations. Indeed, the observed pattern differs from that predicted under simple genetic drift, i.e., greater differentiation among small, isolated (peripheral), populations than larger, more interconnected (core) populations [8]. However, the reduced variance observed in marginal populations could also reflect the generally lower levels of climatic variation across survey sites in the most westerly and easterly bioregions (Broken Hill Complex and COAST bioregions respectively) compared with those towards the centre of *E. plantagineum*'s distribution. Clearly, further data are required to resolve whether convergence in seed mass among range-edge populations has occurred as a result of selection, other demographic or evolutionary processes, or simply as a sampling artefact.

From an evolutionary standpoint, the development of clinal population-level variation in fitness-related traits observed in invasive species both here and globally (e.g., [11],[12],[16],[22],[36],[75],[76]; although see [25] for a contrasting example) can arise via several different mechanisms. Clinal differentiation can occur via adaptive radiation, the evolution of diversity within a rapidly expanding lineage or during range expansion as a restricted number of founder genotypes incrementally diverge over time [9],[16]. This process can be facilitated by an admixture of populations sourced from different parts of the native range and generations of novel genotypes for selection to act upon [11],[16],[34]. Alternatively, a broad base of genotypes may be introduced across the species range, mean trait shifts occurring as a result of selective filtering of pre-adapted or climatically matched genotypes [9],[16]. While the latter process is sometimes not seen as adaptive evolution [9], it does still involve incremental improvement in population fitness via natural selection of phenotypes with heritable traits, which is a condition necessary for evolutionary change [77].

In *E. plantagineum*, both processes have probably taken place, as is the case in invasions more generally [16]. Early introductions of *E. plantagineum* are thought to have come from a variety of areas in the native range, including England, Morocco and France, with multiple introductions occurring across eastern Australia in the mid- to late 1800's [51]. Between 1910 and 2010 populations expanded and merged (Fig. 1a), and during this time significant mixing of genotypes has been likely. Populations in Australia are extremely genetically diverse, recombinants are ubiquitous [50], and overall levels of genetic diversity are similar to that observed in the native range [72]. The breeding system of *E. plantagineum* has also diverged in Australian and native range populations [66] with Australian populations being self compatible and able to outcross [50]. These lines of evidence suggest that the clinal development observed in *E. plantagineum* in this study can be at least in part be explained by evolutionary adaptive radiation and not simply by fitness optimisation of populations via filtering of pre-adapted genotypes.

The rate at which adaptive variation in seed mass exhibited by *E. plantagineum* in Australia has developed is noteworthy. Mean differences in seed mass of around 25% have occurred in, at the very most, 150 generations, which is towards the lower end of the evolutionary rates observed, in traits related to invasiveness, elsewhere [5],[6],[9],[10],[16],[78]–]81]. Despite this short timeframe, geographic patterns in seed mass observed in *E. plantagineum* have apparently converged with those observed in other native Australian forbs.

The results of this study have significant long-term management implications for *E. plantagineum* and other invasive species globally. Predicted global climate change is expected to favour species that have the capacity to rapidly adapt to new conditions [36], and these are the same characteristics that facilitate the invasion of new environments [82]. Our study supports the view that the fitness and range potential of invasive species can rapidly increase as a result of genetic divergence of populations along broad climatic and geographic gradients, and that selection for seed mass can play in important role in this process.
